# Can too many copies spoil the broth?

**DOI:** 10.1186/1475-2859-12-128

**Published:** 2013-12-20

**Authors:** Rochelle Aw, Karen M Polizzi

**Affiliations:** 1 Department of Life Sciences, Imperial College London, London SW7 2AZ, UK; 2 Centre for Synthetic Biology and Innovation, Imperial College London, London SW7 2AZ, UK

**Keywords:** *Pichia pastoris*, *Komagatella phaffi*, Multi-copy clones, Genetic instability, Unfolded protein response, Secretion saturation

## Abstract

The success of *Pichia pastoris* as a heterologous expression system lies predominantly in the impressive yields that can be achieved due to high volumetric productivity. However, low specific productivity still inhibits the potential success of this platform. Multi-(gene) copy clones are potentially a quick and convenient method to increase recombinant protein titer, yet they are not without their pitfalls. It has been more than twenty years since the first reported use of multi-copy clones and it is still an active area of research to find the fastest and most efficient method for generating these strains. It has also become apparent that there is not always a linear correlation between copy number and protein titer, leading to in-depth investigations into how to minimize the negative impact of secretory stress and achieve clonal stability.

## Review

## Background

*Pichia pastoris* has, in recent years, become one of the more popular platforms for heterologous protein expression, surpassing *Saccharomyces cerevisiae* as the preferred yeast recombinant expression system [1]. The popularity of *P. pastoris* stems from high volumetric productivity, resulting in cell densities up to 130 g L^-1^, with a lack of fermentative products [2] and a more favorable glycosylation pattern with N-linked oligosaccharides chains of no more than 20 links [3]. However, despite these high cell densities low specific productivity is still an issue, resulting in extensive research to improve titer levels. One of the best established methods for increasing titer is to increase the number of cognate genes with the intention that this will lead to an increase in transcription and translation of the desired gene. Theoretically, a clone with two identical copies of a gene under the control of an identical promoter should produce twice as much protein. In practice however, the results are more mixed and in many cases the actual titer is below what would be predicted from the number of genes inserted (see below).

Regardless of the mixed success of multi-copy clones, many groups continue to use this as a key strategy for increasing heterologous protein yield. The research into the development of multi-copy strains can focus on two areas. The first (and easiest) experiments look at the quickest and most efficient way of generating multi-copy strains and whether or not this is successful in increasing yield for the particular protein being expressed. The second strategy investigates the effect of these multi-gene copies on the physiology of the cell, including stress on the secretory pathway and potential genetic instability.

### Generating multi-copy clones

Depending on the design and method of transformation, it is possible to integrate multiple copies of a heterologous gene into the *P. pastoris* genome.

**Figure 1:**
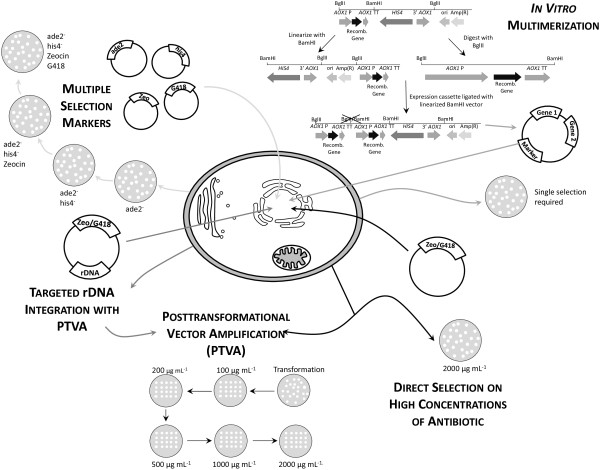
**Methods to generate multi-copy clones.** Schematic representation of some of the more common methods used to create multi-copy clones. **Multiple selection markers** can be used when a gene is integrated into the genome through a vector with a single selection marker. This method is limited to the number of selection markers available (either antibiotic or through complementation to auxotrophic genes). Additionally, each vector must be transformed sequentially and the labor associated with selection increases with each additional gene. ***In vitro multimerization*** uses the pAO815 vector that isolates an expression cassette containing the promoter, gene of interest and transcription terminator region and ligating this in a head-to-tail orientation into a linearized vector. Copy number is determined prior to integration into the genome. **Direct selection on high concentrations of antibiotic** uses a single transformation with a vector containing either G418 or Zeocin™ and selection directly onto high concentrations of the antibiotic. This results in jackpot colonies (over 10 copies of the gene) in less than 1% of all clones. **Posttransformational vector amplification (PTVA)** uses a single vector for transformation (containing either the G418 or Zeocin™ resistance marker). Selection is originally on a low concentration of the corresponding antibiotic, but the cells are increasingly subjected to higher concentrations. Only colonies that have multiple copies of the resistance gene (and therefore multiple copies of the heterologous gene) will be able to survive on the highest concentrations. Jackpot colonies are reported in 6% of all clones tested. **Integration into the rDNA locus with PTVA** utilizes the repeat sequence of the rDNA (appearing 16 times in GS115), which can prevent tandem head-to-tail integration. Multi-copy clones are generated using PTVA.

One of the easiest methods for generating a strain carrying multiple cognate genes is to use different selection markers for sequential integration (Figure 1, top left) [4]. This will require repeat transformations, each with a different selection marker. The drawback of this method is that copy number can only correlate to the number of selection markers available (either antibiotic or auxotrophic markers) and the cost can increase significantly if multiple antibiotics are used.

Gene insertion through a single crossover event can lead to multiple copies being integrated in approximately 1% of all transformants, often in a head-to-tail orientation [4]. These strains can be identified through extensive screening; however this can often be labor intensive. The use of antibiotics, such as Geneticin® (G418) and Zeocin™, and their corresponding antibiotic resistance genes can decrease the workload associated with screening for multi-copy clones. The user is able to select multiple integrants by modulating the antibiotic concentration (Figure 1, bottom right), something which cannot be done when using auxotrophic markers for selection. Some of the first studies that created multi-copy clones used a two-step selection method to identify multiple integrants [5-7]. Initial selection for clones containing the Geneticin® resistance gene was carried out using complementation to *HIS4* and selection on medium lacking histidine. A second round of selection was then carried out using plates containing G418 at varying concentrations (0.5, 1.0, 1.5, 2.0 mg L^-1^) [8]. Theoretically, only clones with increased copies of the antibiotic resistance gene would be able to survive on the higher concentrations of G418. The development of vectors containing a fully functional *Tn903kanr* gene allows for selection directly onto G418, which decreases the workload involved. Furthermore, this reduces the vector size as the *HIS4* gene is no longer required; thereby increasing transformation efficiency as smaller vectors integrate more frequently into the genome [9]. Along with the modified *Tn903kanr,* the increasingly prevalent use of the Zeocin™ resistance gene as a selection marker (where one-step selection is the norm) makes creating multi-copy clones easier [10].

Due to the popularity of multi-copy clones, commercial kits are available that aim to increase the number of heterologous genes integrating into the genome. From the commercial kits, the idea of *in vitro* multimerization before genomic integration has developed (Figure 1, top right). The in vitro method uses the pAO815 plasmid to amplify only the expression cassette (promoter, gene of interest, transcription terminator) [11], so that the entire expression construct will only have one copy of the *HIS4* gene, origin of replication and ampicillin resistance marker (for selection in *Escherichia coli*) [12]. The disadvantage of this method is that it results in a large plasmid, which is notoriously difficult to transform successfully into *P. pastoris*[9]. Thus, there is a limit to the number of copies of the gene of interest that can be integrated using this approach. A modified version of this procedure can be implemented using the popular pPICZ plasmid and its Zeocin™ resistance marker. This may be a more advantageous strategy, as it has been noted that the use of *HIS4* as a selection marker does not always result in stable integration [4].

An alternative commercial product relies on a color-based selection marker, which does not increase the probability of multiple cognate genes but instead makes the screening process easier. This system utilizes an *ADE2* auxotroph strain, where the color of the colonies relates to the copy number [13]. The *ADE2* gene catalyzes the sixth step in the production of purine nucleotides by encoding phosphoribosylaminoimidazole carboxylase [14] and a mutation in *ADE2* will lead to an accumulation of purine precursors, resulting in a red color. Therefore, the more copies of *ADE2* that are complemented into the genome, the less purine precursors that will build up and the whiter the colony will appear.

In 2008, Sunga *et al*. described a new method for the production of multi-copy clones, posttransformational vector amplification (PTVA, Figure 1, bottom center) [15]. PTVA works on the basis that increasing the concentration of antibiotic (particularly Zeocin™ or G418) in a stepwise manner results in an increase in the percentage of multi-copy clones and, in particular, “jackpot” clones with more than 10 copies of the integrated vector. From an initial transformation, single colonies are selected from a plate containing 100 μg mL^-1^ Zeocin™. These are then spotted onto another Zeocin™ plate at the same antibiotic concentration and left to grow for 3–5 days. Each spot is then replicated onto another plate containing increasing concentrations of Zeocin™ (e.g. 200, 300, 500, 1000 and 2000 μg mL^-1^ Zeocin™ were used in the original paper). Since Zeocin™ resistance is obtained by the production of a protein that sequesters the antibiotic (rather than an enzyme which catalyzes its degradation), resistance to higher concentrations of Zeocin™ requires an increased amount of resistance protein. The theory behind PTVA is that increased levels of resistance protein will be due to increased copies of the entire vector [15].

PTVA has been adopted by other groups, with one reporting a total of 52 copies of the *PIP* gene integrated through this method [16]. Additionally, PTVA has also been adapted for integration into the ribosomal DNA (rDNA) locus [17], which has 16 identical repeat regions in *P. pastoris*[18], any or all of which can serve as a site for integration (Figure 1, bottom left).

### The unpredictability of multi-copy clones

**Figure 2:**
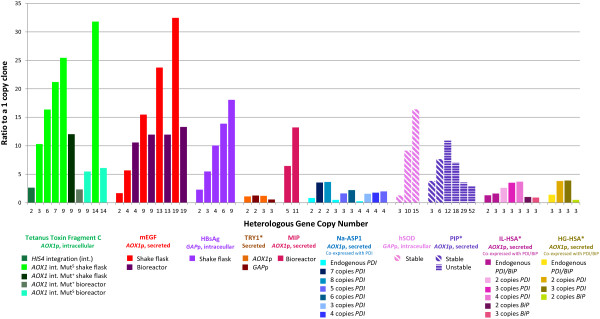
**The impact of multi-copy clones on titer levels.** Expression or activity levels were determined from published data and are presented as a ratio compared to the expression or activity of a single copy strain (calculated by dividing by the equivalent value of a single copy clone). A star (*) indicates that values were estimated. Tetanus toxin fragment C utilized different integration sites (*HIS4* or *AOX1*) by linearizing the vector with different restriction sites prior to transformation. Intracellular expression using the *AOX1* promoter. Samples were grown in either shake flasks or bioreactors [5]. Mouse epidermal growth factor (mEGF) was expressed as a secreted protein using the *AOX1* promoter. Samples were grown in shake flasks and bioreactors [19]. Hepatitis B surface antigen (HBsAg) was expressed intracellularly under the *GAP* promoter in shake flasks [12]. Trypsinogen (TRY1) was expressed extracellularly using the *GAP* promoter or *AOX1* promoter [20]. Miniproinsulin (MPI) was expressed using the *AOX1* promoter as a secreted protein [21]. *Necator americanus* secretory protein (Na-ASP1) was co-expressed with varying copies of protein disulfide-isomerase (PDI) to determine the impact of chaperone coexpression. All variants were secreted and expressed under the *AOX1* promoter [22]. Human superoxide dismutase (hSOD) was expressed intracellularly under the *GAP* promoter. Integration occurred at the rDNA locus with multi-copy clones generated by PTVA. Stability was observed for 28 generations, indicated by the diagonal stripes [17]. Porcine insulin precursor (PIP), using PTVA to generate multi-copy clones, was expressed under the *AOX1* promoter and secreted [16]. Instability was observed in clones (under inducible conditions) with a copy number above 6, indicated by the horizontal stripes [23]. Interleukin and human growth hormone proteins were fused with HSA, IL-HSA and HG-HSA respectively, and co-expressed with PDI or BiP. The fusion proteins were secreted under the control of the *AOX1* promoter [24].

Multi-(gene) copy strains have been used since the early 1990s [4,19]. Figure 2 summarizes the results from a wide range of investigations where multi-copy clones were generated and analyzed. It is apparent that while in many cases an increased copy number results in an increase in product, the relationship is not always linear and there are also instances where a higher copy number leads to a reduction in heterologous protein. Based on the studies to date, the choice of promoter, the presence of a secretion signal and the strain utilized can have an impact on the feasibility of using multi-copy clones for increased titer production.

The first use of multi-copy clones was in 1991 by Clare *et al*. in the production of tetanus toxin fragment C as an intracellular protein [5]. In this study, increasing the number of gene copies from a single copy to 14 copies resulted in a 10% increase in heterologous protein yield [25]. The production of the heterologous protein relied on the alcohol oxidase (*AOX*) 1 promoter, which has been noted to impact the success of multi-copy clones. With this promoter, two types of integration are possible. A double crossover event removes the native *AOX1* gene, resulting in a strain with the methanol utilization slow (Mut^S^) phenotype. A single crossover, preserves the *AOX1* gene, resulting in a Mut^+^ phenotype [25]. In the Mut^S^ phenotype, growth will be significantly reduced on methanol, which could make more resources available for protein production. The study by Clare *et al*. evaluated the impact of both methanol utilization phenotypes when expressing tetanus toxin fragment C and found lower yields in the Mut^+^, supporting this hypothesis (Figure 2) [5].

Vassileva *et al*. found a significant linear relationship (R^2^ = 0.98) between gene dosage and titer in the production of hepatitis B surface antigen (HbsAg), with four copies resulting in a four-fold increase in yield [26]. This was under the control of the constitutive glyceraldehyde-3-phosphate dehydrogenase (*GAP*) promoter, which is significantly weaker than the *AOX1* promoter. It is possible that reduced burden on the cell may account for the linear correlation. Additionally, Marx *et al*. reported that for the production of human Cu/Zn superoxide dismutase (hSOD) integrated in to the rDNA locus, there was a strong correlation between titer and copy number [17]. In each case, the proteins were expressed intracellularly, which prevents additional stress on the secretory pathway and perhaps explains the relationship between increasing gene copy number and higher titer.

In many industrial settings producing the protein as a secreted product is desirable in order to ease downstream processing. Therefore, it is important to examine the effect of protein secretion on the yield obtained from multi-copy clones. Clare *et al*. expressed the mouse epidermal growth factor (mEGF) as a secreted protein using the *AOX1* promoter [19]. For growth in both shake flasks and bioreactors, titer increases with increasing copy number, although this is less prominent when the strains are grown in bioreactors (Figure 2). An increase in titer with multi-copy clones was also observed when expressing miniproinsulin (MPI) extracellularly under the control of the *AOX1* promoter. A direct correlation between copy number and titer was observed, with five copies of the *MPI* gene resulting in a six-fold increase relative to a one copy clone and an 11-copy clone resulting in a 13-fold increase [21].

Nevertheless, such direct correlations between copy number and titer have not always been observed [20]. Proteins targeted to the secretory pathway often show evidence of secretion saturation, whereby increasing gene copy number does not always equate to higher titer [20]. Although secretion saturation does not occur at the same copy number for different proteins (most likely due to the stability of the individual protein and the difficulty of folding [27]), the trend of plateauing yield is the same. When Marx *et al*. expressed secreted human serum albumin (HSA), only clones with up to seven copy numbers showed a similarly high correlation. After this, titer plateaued irrespective of the number of genes integrated [26]. Inan et al. expressed *Necator americanus* secretory protein (Na-ASP1) and found titer capped at three copies [22]. Further evidence of increasing copy number having a detrimental effect on yield was also demonstrated by Zhu *et al*., who determined that an increase in copies of the porcine insulin precursor (*PIP*) up to 12 copies had a positive effect on titer but thereafter any further increase in copy number resulted in not only a reduction in titer but, also, a reduction in growth [16].

### Biochemical basis of reduced yield in multi-copy clones

The evidence for secretion saturation raises a fundamental concern about the impact of increased traffic through the secretory pathway. In 2004, Hohenblum *et al*. [20] compared the response of a recombinant strains with one, two or three copies of the *TRY1* gene under different promoters. It was noted that increasing copy number did not increase titer when using the *GAP* promoter, but when using the *AOX1* promoter, titer increased from a single copy clone to two copies and then reduced dramatically (to levels lower than with a one copy clone) with a three copy clone (Figure 2). As *AOX1* is a highly active promoter, Hohenblum *et al*. suggested that the amount of protein produced was causing stress on the secretory pathway, leading to the upregulation of the unfolded protein response (UPR) and resulting in degradation of the protein. [20,28]. The UPR is a signaling pathway activated in response to the accumulation of unfolded proteins in the endoplasmic reticulum (ER). UPR activation results in the upregulation of a number of genes, with the ultimate aim of restoring ER homeostasis. An excess of unfolded or misfolded protein causes the dissociation of Kar2p (also known as BiP) from Ire1p and allows these membrane proteins to oligomerize [29]. The Ire1p, which is an ER-located transmembrane kinase and endoribonuclease, then initiates the splicing of *HAC1* mRNA, which activates the upregulation of key UPR genes [30-32]. Among these are chaperones such as Kar2p and protein disulfide-isomerase (PDI), which increase the folding capacity in the ER [33]. The UPR has severe negative impacts on protein production. Under prolonged signaling, the ER-associated degradation (ERAD) pathway is activated [34,35], resulting in retrotranslocation of misfolded protein to the cytosol for degradation by the proteasome [36-39], reducing the overall yield. Proteins that are poor folders are more likely to induce the UPR and to activate the ERAD, which is one explanation as to why secretion saturation is reached at different copy numbers in different systems [27]. A simple way to improve yield is to fuse the protein of interest with a protein that folds and secretes well, such as HSA [24,40,41]. Because HSA can reach such high titers (up to 10 g L^-1^), the impact on the secretory pathway is significantly reduced, as translation is not impeded by the need to produce additional chaperones to help with folding [27]. By increasing the ease through which the proteins can move through the secretory pathway, this will theoretically prevent the upregulation of the UPR [42,43].

Attempts to control the UPR have also been investigated in an effort to alleviate cellular stress and increase protein production [32,44]. One such method is the co-expression of chaperone proteins alongside the protein of interest, which has been reported to reduce the effects of the UPR. In single copy clones of *P. pastoris*, it has been reported that synergistically expressing Kar2p/PDI can increase secretion levels by up to 6.5 times [45]. When using multi-copy clones, co-expressing PDI alone appears to result in a consistent increase in protein titer. However, co-expressing Kar2p alone is a less successful strategy (Figure 2) [24]. Inan *et al*. showed that there was a strong correlation between the number of copies of *PDI* integrated into a strain and the amount of secreted Na-ASP1. However, there was still evidence of intracellular accumulation of Na-ASP1 regardless of the number of *PDI* genes included, suggesting that overexpressing a chaperone does not completely overcome the blockage in the secretory pathway [22]. Another strategy has been to overexpress the main transcriptional regulator of the UPR, Hac1p, which has been successfully demonstrated in *S. cerevisiae* and *Aspergillus niger* var. *awamori*[46,47]*.* In *P. pastoris* overexpression of Hac1p in a single copy strain yielded mixed results depending on the heterologous protein in question [30].

### The problem of strain stability

**Figure 3:**
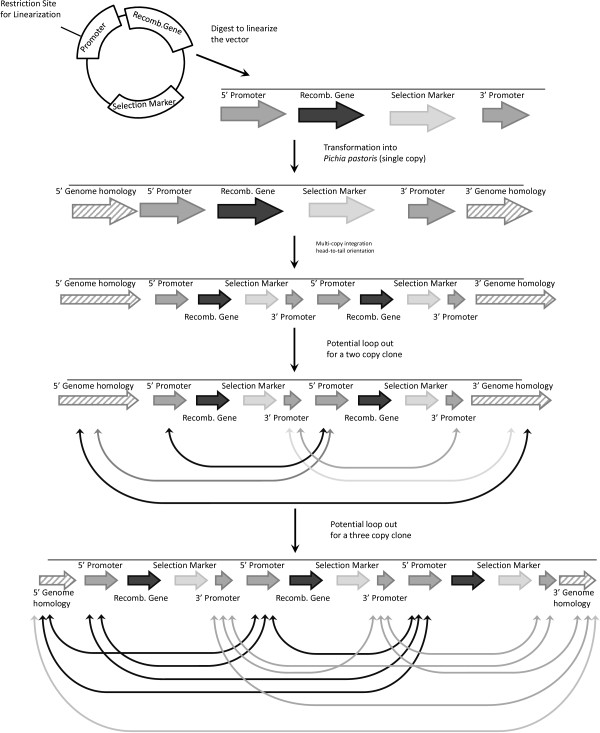
**Loop out recombination.** Through the highly recombinogenic nature of *P. pastoris* multiple copies of the vectors can integrate in a head-to-tail orientation. This will create repeat regions of homology which can recombine to remove either the whole or parts of the vector. For a two copy clone there is the potential for at least five loop out regions (based on the design of the vector) and this can increase to at least 11 for a three copy clone.

In addition to secretion saturation, there have been a handful of papers that have made reference to the problems of genetic instability in multi-copy clones. One of the advantages of using *P. pastoris* is the ease with which it can be genetically modified [2]. Nevertheless, it is perhaps this highly recombinogenic nature that results in unstable clones. Theoretically, an organism that so readily accepts DNA can lose it just as fast. In 1998, Ohi *et al*. reported that a clone with two copies of *HSA* integrated into the *HIS4* locus and grown for 163 hours (83 generations) resulted in 0.1% of cells losing the foreign gene [48]. In both *S. cerevisiae* and *Yarrow lipolytica*, it was determined that the generation of multi-copy clones through tandem integration could result in the excision of the integrated genes through a loop-out method (Figure 3) [49-52]. As integration in *P. pastoris* occurs through similar mechanisms, the assumption is that recombination of this variety can also occur.

In both *S. cerevisiae* and *Y. lipolytica* integrating into the rDNA locus has been shown to prevent recombination between homologous regions, resulting in higher copy number stability [50,53]. The same integration into the rDNA locus was established in *P. pastoris* in 2006 by Steinborn *et al*. [54]. While in *S. cerevisiae*, the number of rDNA repeats ranges from around 100–200 copies [55], this is significantly reduced in *P. pastoris* GS115, with only 16 copies identified. Thus, the number of independent integrations that can occur at unique rDNA loci is also reduced [18]. In 2009, Marx *et al*. combined rDNA locus-directed integration with PTVA to generate multi-copy clones and determined that these were stable for up to 28 generations (Figure 2) [17].

In the same year, Zhu *et al*. undertook one of the most intensive investigations into the stability of multi-copy clones to date, after generating a wide range of strains with varying copy number through PTVA [23]. Real-time quantitative PCR was used to determine the copy number of clones containing 1, 6, 12, 19 and 29 copies of the *PIP* gene, both pre-induction (growth on glycerol) and post induction (growth on methanol). It was determined that clones with less than six copies of the heterologous gene were stable but any clones with higher copy numbers showed copy number instability when induced with methanol (Figure 2). Stability was observed for all clones when grown on a glycerol-based medium.

To combat the highly recombinogenic nature of *P. pastoris,* Näätasaari *et al*. have developed the *ku70* deletion strain to prevent non-homologous recombination by knocking out the main protein responsible for non-homologous end joining in double-stranded DNA break repair [56]. This method intends to prevent the vector from integrating anywhere other than the desired target location. However, Näätasaari *et al*. also show that the *ku70* deletion strains have increased stability, as no changes in copy number were observed after 72 hours of methanol induction when the protein was expressed intracellularly. Despite this, it is important to note that the maximum number of copies used in this investigation was eight, whereas the first copy number to exhibit instability in the systematic investigation by Zhu *et al*. was 12, suggesting that it would be of interest to determine the stability of strains with higher copy numbers. It would also be of interest to determine whether secretory stress impacts the stability of the strain by expressing secreted proteins in this strain.

### Conclusions and future perspectives

The draw of increasing titer through the use of multi-copy clones is so enticing that, despite having originally been described over 20 years ago, it is an aspect that is still being investigated in depth today. The fact that the choice of promoter, strain, method of expression and heterologous protein of interest can impact the titer produced by multi-copy clones implies that these variables may have an impact on the stability of the strain. With proteins expressed intracellularly, there will be no stress on the secretory pathway; thus preventing the impact of the UPR. Theoretically, if the cells are not undergoing such an intense cellular stress then natural selection will not force the selection of clones with a lower copy number. It is important to note that recombination between regions of homology is likely to still occur but the propensity for lower copy clones may not be as high. However the advantage of *P. pastoris* as a heterologous expression platform lies in the reduced downstream processing required for secreted proteins due to the minimal amount of native proteins expressed. Thus, it is vital that improvements to the functionality of multi-copy clones with extracellular expression are developed.

The advent of better selection markers and more stable strains, as well as the implementation of corresponding methods that have been effective in other yeast, make the possibility of creating stable multi-copy clones more promising. Several additional strategies to decrease instability and/or secretory stress could be explored in the future. For example, it has been reported in *S. cerevisiae* that integrating into the transposable element *Ty1*, present in approximately 30 to 40 copies within the genome, results in even higher stability than using the rDNA locus [51,52]. It would be interesting to investigate whether there is an equivalent *Ty1* gene that can be used for generating more stable multi-copy clones in *P. pastoris*.

The current work aiming to modulate the upregulation of the UPR may turn out to be a critical aspect for improving yields from multi-copy clones. Many groups have investigated ways to reduce the effects of the UPR, predominantly by co-expressing chaperones such as PDI or Kar2p, e.g. [22,57] or by overexpressing Hac1p [44]. However, these approaches are often protein specific and do not work in a general scheme; thus the method has to be individually tailored for the particular protein in question. Ultimately, it may be necessary to consider a more general strategy that can be applied to all proteins of interest.

One such strategy is to consider ER expansion, a method that has been evaluated in *S. cerevisiae*[58]. ER expansion has been proven to independently alleviate UPR stress in the absence of co-expressing folding chaperones. If homologs of the relevant proteins (ino2/4) can be determined in *P. pastoris*, then increasing the size of the ER may mean that secretion saturation is reached at a higher capacity, resulting in increased titer for all proteins. Theoretically, a cell that is able to deal with additional nascent protein without inducing the UPR, could achieve higher yields.

Another potential generic method for increasing protein yield was discussed in a 2013 paper by Larsen *et al*., which identified “super-secretor” phenotypes of *P. pastoris* that, independent of the recombinant proteins expressed, resulted in increased titer [59]. One such mutant strain, *beta-galactosidase supersecretion* (*bgs*) 13, was identified as a key strain that could prove to be pivotal in increasing the capacity for protein expression. Out of five heterologous proteins tested, *bgs13* knockout strains showed increased protein production for four of the proteins. As all of the *BGS* genes identified are not related to the secretory pathway, it suggests that these strains are increasing titer by manipulating other factors. It would be of interest to observe how these strains would behave with multiple gene copies and whether stress on the secretory pathway was still observed.

For multi-copy clones to be fully utilized, further research is required to ensure that methods to generate these strains are quick and effective, the resulting strains are stable and that the cell is capable of dealing with the additional heterologous proteins. The story of multi-copy clones is already more than twenty years old, but it is far from over. The potential of this method can only be fully discovered as researchers attempt to understand more about the basic toolbox of *P. pastoris*, perhaps something that has been ignored for far too long.

## Abbreviations

AOX1: Alcohol oxidase; BGS: Beta-galactosidase supersecretion; ER: Endoplasmic reticulum; ERAD: ER associated degradation pathway; GAP: Glyceraldehyde-3-phosphate dehydrogenase; HbsAg: Hepatitis B surface antigen; HG-HSA: Human growth hormone fusion protein with HSA; HSA: Human serum albumin; hSOD: Human Cu/Zn superoxide dismutase; IL1ra: Interleukin-1 receptor antagonist; IL-HSA: IL1ra fusion protein with HSA; mEGF: Mouse epidermal growth factor; MPI: Miniproinsulin; MutS: Methanol utilisation slow; Na-ASP1: *Necator americanus* secretory protein; PDI: Protein disulfide-isomerase; PIP: Porcine insulin precursor; PTVA: Posttransformational vector amplification; rDNA: Ribosomal DNA; TRY1: Trypsinogen; UPR: Unfolded protein response.

## Competing interests

The authors declare that they have no competing interests.

## Authors’ contributions

RA conceived of the manuscript and helped draft the manuscript. KP helped draft the manuscript. Both authors read and approved the final manuscript.
